# Supplementary material for: Roles for PKC signaling in chromaffin cell exocytosis
Source: Biophys J. 2024 Dec 4;124(11):1785–97. doi: 10.1016/j.bpj.2024.12.005 (PMC12256897; doi:10.1016/j.bpj.2024.12.005)
Supplement: Document S1. Figures S1–S3 [file mmc1.pdf]

Biophysical Journal, Volume *124*

Supplemental information

Roles for PKC signaling in chromaffin cell exocytosis

Xiaohuan Chen, Nicole A. Bell, Breanna L. Coffman, David R. Giovannucci, and Arun Anantharam

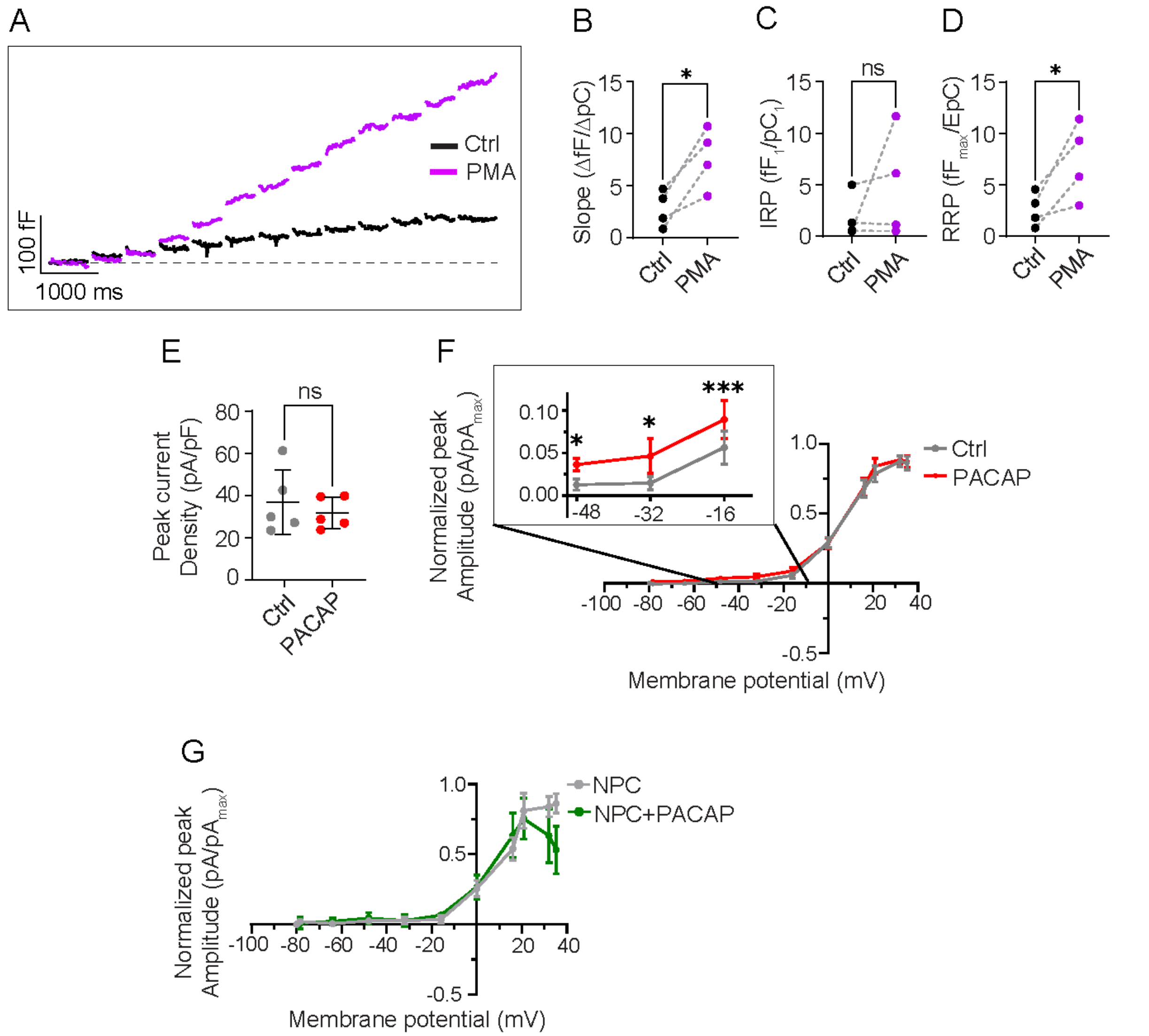

Figure S1: Electrophysiological studies on the effects of PMA and PACAP on chromaffin cell excitability and secretion.

PKC effects on activity-dependent exocytosis were measured using the previously described capacitance paradigm to estimate Ca^{2+} sensitivity, IRP and RRP. PMA was bath applied for 1 minute following a control train and maintained during the second pulse train (A) An example trace of measured capacitance changes (ΔC_m) between a control (black trace) and the PKC

agonist phorbol 12-myristate 13-acetate (PMA) (0.1 μM) (purple trace) treated cell. (B) A comparison of the $pC/\Delta C_m$ relationship between cells pretreated with or without PMA (0.1 μM). Ca^{2+} sensitivity of exocytosis appeared enhanced between the control and PMA (0.1 μM) treated cells ($p = 0.0192$). (C) There was no change in IRP size of PMA treated cells ($p= 0.6$). (D) There was a significant increase in the RRP measured for PMA-treated cells ($p = 0.04$) (n=4). (E) To determine whether PACAP caused an enhancement in Ca^{2+} entry, the peak current density (pA/pF) of Ca^{2+} currents was measured in response to a 50 ms step-depolarization from -80mV to +20mV prior to and following PACAP treatment. Incubation of PACAP (0.5μM) for 1 min did not significantly affect the Ca^{2+} entry ($p = 0.3$) (n=5). (F) To assess PACAP effects on Ca^{2+}channel activation, i-v relationships were measured using a voltage ramp protocol prior to and following PACAP treatment. Following transformation of ramp duration to changes in membrane voltage, the average Ca^{2+} current response to voltage ramp-depolarization from -80mV to +80mV for 100 ms intervals in chromaffin cells were normalized to respective peak amplitude (pA/pA_{max}) and compared. The averaged data showed a modest PACAP-mediated enhancement Ca^{2+} current at membrane potentials of -48 mV ($p = 0.03$), -32 mV ($p = 0.02$), and -16 mV ($p = 0.0005$). Cells were incubated in PACAP (0.5μM) for 1 min prior to test ramp application (n=12). (G) To assess if PACAP-mediated effects on Ca^{2+} channels was dependent on PKC activity, NPC (10μM) was applied for 1 min prior to the first voltage ramp depolarization, followed by a 1 min application of both PACAP (0.5μM) and NPC (10μM) applied together for the second voltage ramp depolarization in the same manner as previously described. Current responses were normalized to peak amplitude (pA/pA_{max}) and demonstrated that PACAP-mediated enhancements at -48 mV ($p = 0.2$), -32 mV ($p = 0.2$), and -16 mV ($p = 0.5$) were blocked by NPC (n=4).

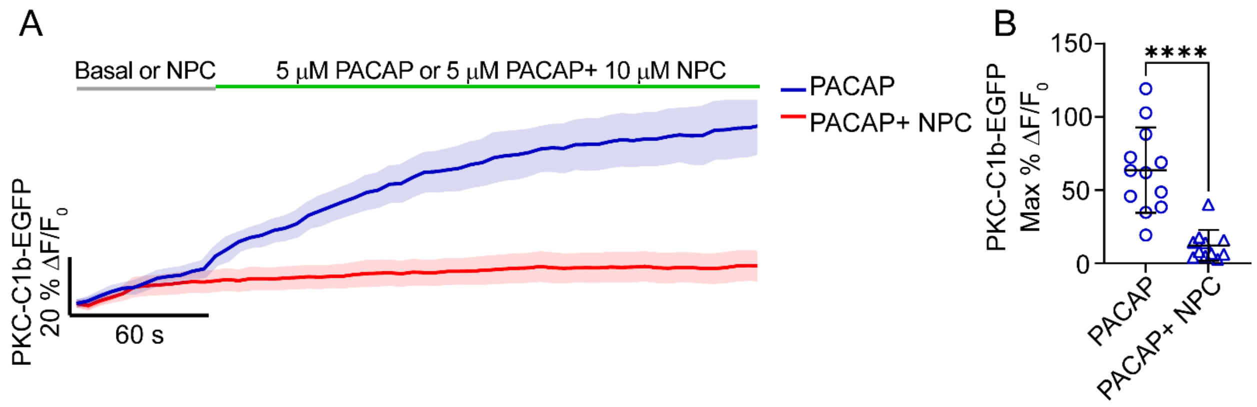

Figure S2. NPC 15437 inhibited PACAP-stimulated translocation of PKCβ C1b-EGFP to the plasma membrane. (A) Time course of PKCβ C1b-EGFP fluorescence changes (%$\Delta F/F_0$) in chromaffin cells stimulated with PACAP (5 µM) alone (blue) or PACAP + NPC 15437 (NPC, 10 µM; red). Cells were pre-incubated in either a basal solution (physiological saline) or with 10 µM NPC for 1 minute, followed by stimulation with PACAP or PACAP + NPC for 4 minutes. Bold lines represent mean fluorescence changes, and shaded areas indicate SEM. (B) Scatter plot showing the maximum %$\Delta F/F_0$ for individual cells under each condition. Data are derived from two independent experiments (PACAP: n = 12 cells; PACAP + NPC: n = 13 cells). Statistical analysis was performed using a two-tailed unpaired t-test, with statistical significance denoted as ****$p < 0.0001$.

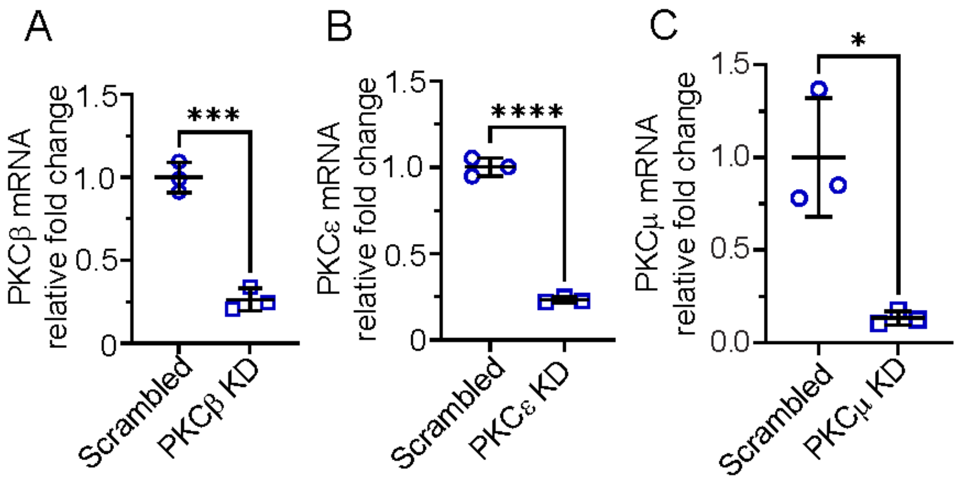

Figure S3. qPCR analysis shRNA knockdown efficiency. qPCR results confirm that shRNAs targeting PKCβ, PKCε, and PKCμ effectively reduce mRNA expression levels by at least 70% for each isoform. Specifically, (A) PKCβ knockdown reduces mRNA by 73%, (B) PKCε by 77%, and (C) PKCμ by 87%. Data are presented as relative fold change compared to scrambled shRNA controls.
