# Supplementary material for: Roles for PKC signaling in chromaffin cell exocytosis
Source: Biophys J. 2024 Dec 4;124(11):1785–97. doi: 10.1016/j.bpj.2024.12.005 (PMC12256897; doi:10.1016/j.bpj.2024.12.005)
Supplement: Document S2. Article plus supporting material [file mmc2.pdf]

Biophysical Journal
Article

Biophysical Society

Roles for PKC signaling in chromaffin cell exocytosis

Xiaohuan Chen,[1] Nicole A. Bell,[1] Breanna L. Coffman,[1] David R. Giovannucci,[1] and Arun Anantharam[1,*]
[1]Department of Neurosciences, University of Toledo, Toledo, Ohio

ABSTRACT Chromaffin cells of the adrenal medulla have an important role in the sympathetic stress response. They secrete catecholamines and other hormones into the bloodstream upon stimulation by the neurotransmitter pituitary adenylate cyclase-activating polypeptide (PACAP). PACAP causes a long-lasting and robust secretory response from chromaffin cells. However, the cellular mechanisms by which PACAP causes secretion remain unclear. Our previous work showed that the secretory response to PACAP relies on signaling through phospholipase C epsilon (PLCε). The objective of this study was to clarify the role of signaling events downstream of PLCε. Here, it is demonstrated that a brief exposure of chromaffin cells to PACAP caused diacylglycerol (DAG) production—a process that was dependent on PLCε activity. DAG then activated protein kinase C (PKC), prompting its redistribution to the plasma membrane. PKC activation was important for the increases in cytosolic Ca^{2+} and exocytosis that were evoked by PACAP. Indeed, pharmacological inhibition of PKC with NPC 15437, a competitive inhibitor of DAG binding, significantly disrupted the secretory response. NPC 15437 application also eliminated PACAP-stimulated effects on the readily releasable pool size, the Ca^{2+} sensitivity of granule fusion, and the voltage dependence of Ca^{2+} channel activation. Quantitative PCR revealed PKCβ, PKCε, and PKCμ to be highly expressed in the mouse chromaffin cell. Genetic knockdown of PKCβ and PKCε disrupted PACAP-evoked secretion, while knockdown of PKCμ had no measurable effect. This study highlights important roles for PKC signaling in a highly regulated pathway for exocytosis that is stimulated by PACAP.

SIGNIFICANCE °Adrenal chromaffin cells serve a crucial function in the sympathetic stress response by releasing catecholamines and peptide hormones into the bloodstream. Pituitary adenylate cyclase-activating polypeptide (PACAP) has emerged as a key regulator of chromaffin cell secretion. However, the mechanisms by which PACAP stimulates secretion are not well understood. In this study, we define important roles for protein kinase c (PKC) in the PACAP signaling pathway. Genetic or pharmacological disruption of PKC activity strongly inhibits the PACAP secretory response. These findings provide novel insights into the mechanism of PACAP-stimulated secretion and will further our understanding of the actions of PACAP in other tissues throughout the body.

INTRODUCTION

The adrenal medulla, a core effector of the sympathetic nervous system, plays a critical role in the "fight-or-flight" response by releasing epinephrine, norepinephrine, and a variety of peptide hormones into the bloodstream (1). Chromaffin cells, the secretory units of the adrenal medulla, synthesize, store, and secrete these hormones (1). Once released, adrenomedullary hormones modulate cardiac, pulmonary, and metabolic functions, promoting survival and maintaining internal stability during stress (2–4).

Secretion from the adrenal medulla relies on input from preganglionic sympathetic fibers that reach the gland via splanchnic nerves (5,6). It has been known for many decades that acetylcholine is released from the splanchnic nerves onto chromaffin cells, prompting the secretion of catecholamines (7). It is now understood that splanchnic neurons also contain peptide neurotransmitters that may be coreleased with acetylcholine to further regulate chromaffin cell secretion (8,9). Recent evidence suggests that, among these peptides, pituitary adenylate cyclase-activating polypeptide (PACAP) may play the most significant role (2,10).

PACAP was first identified in 1989 in the ovine hypothalamus and shown to stimulate adenylate cyclase activity in rat anterior pituitary cell cultures. (11). It has since been established as a powerful regulator of the hypothalamic-pituitary-adrenal circuit and is linked to posttraumatic stress

Submitted September 20, 2024, and accepted for publication December 3, 2024.

*Correspondence: arun.anantharam@utoledo.edu

Editor: Sarah Veatch.
https://doi.org/10.1016/j.bpj.2024.12.005

© 2024 The Authors. Published by Elsevier Inc. on behalf of Biophysical Society.
This is an open access article under the CC BY license (http://creativecommons.org/licenses/by/4.0/).

disorder in humans (12–15). PACAP is now recognized as a key neurotransmitter at the splanchnic-chromaffin cell synapse, capable of causing a robust secretory response from chromaffin cells (16,17).

The PACAP signaling cascade for secretion begins with activation of a $G\alpha_s$-coupled PAC1 receptor (18). This is followed by stimulation of adenylate cyclase and cAMP production. We recently showed that phospholipase C epsilon (PLCε) is activated downstream of cAMP (12,17,19). PLCε then hydrolyzes phosphatidylinositol 4,5-bisphosphate (PIP_2) to generate diacylglycerol (DAG) and inositol trisphosphate (IP3) (20). IP3 gates the release of Ca^{2+} from ER stores by activating IP3 receptors (12). The goal of this study was to define the impact of the PLCε-DAG signaling axis on the secretory response to PACAP.

Here, we show that PACAP stimulation caused DAG production in chromaffin cells downstream of PLCε. When DAG production was prevented (i.e., in cells lacking PLCε), PACAP-stimulated secretion was rarely observed. Furthermore, pharmacological inhibition of DAG-stimulated PKC activity—using the inhibitor NPC 15437—disrupted the Ca^{2+} elevations caused by PACAP, and consequently exocytosis. PKC inhibition also eliminated PACAP-dependent effects on the: 1) Ca^{2+} sensitivity of granule fusion, 2) size of the readily releasable pool (RRP), and 3) voltage activation curve of Ca^{2+} channels. Although multiple PKC isoforms are expressed in chromaffin cells, genetic disruption of PKCε and PKCβ expression, but not PKCμ, attenuated PACAP-evoked elevations in Ca^{2+} and secretion. Together, these findings demonstrate a critical role for PKC signaling in the PACAP-stimulated pathway for secretion in the chromaffin cell. Although the precise molecular targets of PKC are not yet known, the results show it to act at multiple loci that influence Ca^{2+} influx and exocytosis.

MATERIALS AND METHODS

Animals

C57BL/6J mice (referred to as "wild-type" or WT) were obtained from the Jackson Laboratory (Bar Harbor, ME). PLCε −/− mice (also referred to as "KO") were generated by Smrcka and colleagues (21). The mice were group-housed with two to five mice per ventilated cage, maintained under a 12-h dark/12-h light cycle with full access to food and water. All animal procedures and experiments were conducted in accordance with the University of Toledo IACUC protocol (400138).

Mouse chromaffin cell preparation

Mouse chromaffin cells were isolated and cultured after previously well-established protocols (19,20), using reagents and cell culture wares from the same sources and catalog numbers as mentioned previously. In brief, 8- to 12-week-old male or female mice were anesthetized with isoflurane and euthanized via cervical dislocation. Adrenal glands were extracted and transferred to dishes containing ice-cold mouse buffer (148 mM NaCl, 2.57 mM KCl, 2.2 mM $K_2HPO_4 \cdot 3H_2O$, 6.5 mM KH_2PO_4, 10 mM glucose, 5 mM HEPES, 14.2 mM mannitol [pH adjusted to 7.2]). After removing the cortex, the medullae were rinsed in a papain solution (450 units/mL papain, 250 μg/mL bovine serum albumin [BSA], and 75 μg/mL dithiothreitol in mouse buffer). They were then transferred to a 15-mL Falcon tube containing 0.5 mL of papain solution and placed in a water bath shaker at 37°C, shaking at 140 rpm for 15 min. The papain solution was then mostly removed and replaced with 0.5 mL of collagenase solution (250 μg/mL BSA, 3.75 mg/mL collagenase, and 0.15 mg/mL DNase I in mouse buffer). Digestion continued for another 15 min under the same conditions. Postdigestion, the medullae were transferred to DMEM/F12 medium supplemented with 10% fetal bovine serum, triturated using a pipette, and centrifuged at 300 × g for 2.5 min. The resultant pellet was resuspended in 300 μL medium. Before cell preparation, 35 mm glass-bottom dishes (10 mm glass diameter) were precoated with Matrigel diluted in DMEM/F12 (1:7) for 1–1.5 h, followed by washing with DMEM/F12. The cells were cultured in an incubator (37°C, 5% CO_2) for approximately 4 h. Then, a culture medium with antibiotics was added to a final volume of 2 mL (DMEM/F12 supplemented with 10% fetal bovine serum, 9.52 units/mL penicillin, 9.52 μg/mL streptomycin, and 238 μg/mL gentamicin). The medium was replaced the day after plating, and experiments were conducted between 18 and 48 h after plating.

Plasmids, transfection, and reagents

The GCaMP5G plasmid was obtained from Addgene (31788). The Lck domain (MGCGCSSNPEDDWMENIDVCENCHYP) was fused to GCaMP5G as described by Shigetomi et al. (22). The neuropeptide Y (NPY) pHluorin plasmid was provided by Dr. Ronald W. Holz (University of Michigan, Ann Arbor, MI). FFN511 (cat. no. ab120331) was purchased from Abcam (Cambridge, UK). The PKCβ C1b domain construct was originally generated in Dr. Alexandra Newton's lab (UCSD) (23). We obtained the C1b-YFP plasmid from Dr. Alan V. Smrcka (University of Michigan). The C1b domain sequence was derived from the mouse PKCβ gene and the YFP was replaced with EGFP for use in this study. Scrambled shRNA with RFP reporter and PKCβ shRNA plasmids were purchased from LipExoGen Biotech (Baltimore, MD). The sequence of scrambled shRNA is 5′-CGTCCTATCAACTCACTGTA-3′, and the sequence of PKCβ (Prkcb) shRNA is 5′-GGTTCTCATTGTTGTTGTAA-3′. Scrambled shRNA with GFP (cat. no. TR30021), PKCε (Prkce) shRNA with GFP (cat. no. TL501656A), and PKCμ (Prkd1) shRNA (cat. no. TL516572A) plasmids were purchased from Origene. PKCε (Prkce) shRNA tagged with mCherry was custom ordered from VectorBuilder. The sequence of scrambled shRNA is 5′-CACAAGCTGGAGTACAACTACAACAGCCA-3′, the sequence of PKCε shRNA is 5′-CTGGCTGACCTTGGTGTTACTCCAGACAA-3′, the sequence of PKCμ shRNA is 5′-GAGATGGCTTGTTCCATCGTGGACCA GAA-3′. Cells were conducted for experiments 48–72 h after transfection, and transfected cells were identified by their fluorescence encoded by the shRNA vectors.

For transfections, cell pellets were resuspended in 110 μL of sucrose-based buffer (250 mM sucrose and 1 mM $MgCl_2$ in DPBS, Gibco, 10010-023). The desired plasmid (1.5 μg/gland) was added to the mixture. The cells were transiently transfected by electroporation with a single pulse (1050 mV, 40 ms) using the Neon transfection system (Invitrogen, MPK5000 and MPK10096). After transfection, the mixture was combined with 200 μL of DMEM/F12 medium and divided into two Matrigel-coated dishes. Four to six hours later, medium with antibiotics was added to the cells up to 2 mL.

In some shRNA knockdown experiments, chromaffin cells were electroporated with shRNA first, and cotransduced with red fluorescent Ca^{2+} sensor R-GECO (Montana Molecular, Bozeman, MT, U0600R), following the manufacturer's protocol. In brief, cells were infected with 20 μL of BacMam DAG sensor stock in 100 μL of medium, supplemented with 2 mM sodium butyrate (Montana Molecular), for 30 min at room temperature, away from light. This was followed by a 4–6 h incubation period in a 37°C tissue culture incubator. After that, medium containing 2 mM sodium

butyrate and antibiotics was added to the cells up to a total volume of 2 mL, and the culture was continued for 48–72 h.

In some cases, cells were not transfected but incubated with 1 μM of the membrane-permeant fluorescent Ca^{2+} indicators Cal-520 AM (AAT Bioquest, Pleasanton, CA, 21130) or Calbryte590 AM (Cal590, AAT Bioquest, 20701) in physiological saline solutions (PSS) (145 mM NaCl, 5.6 mM KCl, 2.2 mM $CaCl_2$, 0.5 mM $MgCl_2$, 5.6 mM glucose, and 15 mM HEPES [pH 7.4]) for 30 min. The NPC 15437 reagent was purchased from Enzo Life Sciences, Long Island, NY (ALX-270-104).

TIRF microscopy

TIRF imaging was performed utilizing an Olympus cellTIRF microscope system (Olympus, Center Valley, PA), configured for two-line (488 nm/561 nm) operation. The microscope was equipped with numerical aperture 1.49 TIRF oil-immersion objectives (60× and 100×) and was sometimes used with an additional 2× lens in the emission path situated between the microscope and the camera (Andor Technology, Belfast, UK, iXon Ultra 897). The resulting pixel size in the images ranged from 80 to 266.7 nm.

All TIRF experiments were executed at a controlled temperature range of 35–37°C, achieved by placing the sample dish on a temperature controller platform (Warner Instruments, Hamden, CT, TC-324C). The culture medium was replaced with warmed PSS 10 min before the experiment. The replacement of the PSS bath was accomplished through a gravity-based system, functioning at a fluid flow rate of 3–5 mL per min. Chromaffin cells were individually stimulated utilizing a 100-μm inner diameter needle (ALA Scientific Instruments, Farmingdale, NY, ALA QTP) in conjunction with a positive-pressure perfusion system (ALA Scientific Instruments, ALA-VM4).

Live cell DAG assay

Chromaffin cells were transduced with a green DAG upward sensor (Montana Molecular, U0300G), following the manufacturer's protocol. In brief, cells were infected with 10 μL of BacMam DAG sensor stock in 150 μL of medium, supplemented with 2 mM sodium butyrate (Montana Molecular), for 30 min at room temperature, away from light. This was followed by a 4–6 h incubation period in a 37°C tissue culture incubator. The BacMam solution was then removed and replaced with DMEM/F12 medium containing 2 mM sodium butyrate for a duration of 18–48 h. Imaging was performed on the TIRF microscope with images acquired every 10 s (100 ms exposure time) for a duration of 5 min with indicated stimulations.

Imaging protocol

Chromaffin cells were stimulated with PACAP (synthesized at University of Iowa by J. Galpin and C. Ahern (19)) for different durations of time. In experiments involving Lck-GCaMP5G, Cal520, Cal590, GECO, NPY-pHluorin, or FFN511 cells were stimulated for 45 s. In experiments involving PKC C1b-EGFP, cells were sequentially exposed to PSS for 1 min, 1 μM PACAP for 2 min, and 5 μM PACAP for 2 min.

Image analysis of Ca^{2+} signal and fusion events

Global Ca^{2+} signals (amplitude, total area of spikes) were measured in cells expressing Lck-GCaMP5G, R-GECO, or loading with Ca^{2+} dye indicators. The frame rate for these experiments was 20 Hz. The region of interest selection was performed in ImageJ; analysis was performed within a custom program written in Interactive Data Language (IDL 6.3). Details of the program are provided in multiple recent studies (19,20,23). For secretion studies, NPY-pHluorin or FFN511 fusion events were visually identified. Regions of interest with a radius of 240 nm were then drawn using the Time Series Analyzer v3.0 plugin on Fiji software. Image acquisition was performed at a frequency of 40 Hz, fusion events were reported as number of events per μm^2 of cell footprint area.

Western blotting

The medulla was isolated from adrenal glands by cutting off the cortex, then lysed in 100 μL of MPER buffer (Thermo Fisher Scientific, Waltham, MA, 78501) supplemented with a protease inhibitor cocktail (Thermo Scientific, 78444). Lysis was carried out on ice for 30 min, with vertexing every 5 min. Lysed samples were centrifuged at 12,000 × g for 15 min at 4°C, and the supernatant was transferred to a fresh tube for protein concentration measurement using a BCA assay (Thermo Scientific, A53227).

Each sample, containing 30 μg of protein, was loaded onto a gradient (4–12%) Bolt Bis-Tris Plus gel (Thermo Scientific, NW04122BOX) for separation in 1× Bolt MES SDS running buffer (Thermo Scientific, B0002) for 2 h at 120 V. Proteins were then transferred to a PVDF membrane (Bio-Rad, Hercules, CA, 162–0177) in 1× Bolt transfer buffer (Thermo Scientific, BT0006) for 1 h at 25 V. The membrane was blocked with 5% BSA in TBST buffer for 1 h at room temperature. Primary antibodies (1:1000) were added and incubated overnight at 4°C. The membrane was washed three times with TBST for 10 min each, then incubated with HRP-conjugated secondary antibodies (1:5000) at room temperature for 1 h. The membrane was washed again three times with TBST for 10 min each. Blots were then developed using an ECL reagent (Thermo Scientific, 34580) in a box western blot imager (G: Box Chemi, Syngene, Karnataka, India).

Antibodies used include PKCβ (Proteintech, Rosemont, IL, 12919-1-AP), PKCε (Proteintech, 20877-1-AP), PKD/PKCμ (Cell Signaling Technology, Danvers, MA, 90039T), β-actin (Invitrogen, Waltham, MA, MA1-140), goat anti-rabbit IgG (H+L) secondary, HRP (Thermo Scientific, 31,460), and goat anti-mouse IgG1 secondary, HRP (Thermo Scientific, A10551).

Real-time PCR

Chromaffin cells isolated from adrenal glands were plated into a 12-well plate culture for 24 h (screen PKC isoforms), or 72 h (knockdown experiments), and were lysed with 500 μL TRIzol reagent (Thermo scientific, 15596026). Following the manufacturer's guidelines, total RNA was extracted and 100 ng of total RNA per sample was converted into cDNA using the SuperScript IV VILO Master Mix kit (Thermo Scientific, 11756500). Quantitative PCR was run with PowerUp SYBR green Master Mix (Thermo Scientific, A25742) on a Quant Studio 3 detection system (Applied Biosystems, Waltham, MA). The $2^{-\Delta CT}$ method was used to quantify relative mRNA expression levels, which were normalized to β actin, the $2^{-\Delta\Delta CT}$ method was used to compare the expression levels between scrambled shRNA and PKCβ, PKCε, and PKCμ shRNA. Custom primers were prepared by Integrated DNA Technologies (Coralville, IA), the sequences of which are specified in Table 1.

Electrophysiological measurements of I_{Ca} and C_m

Standard perforated patch-clamp methods were used to simultaneously evoke and record Ca^{2+} currents while measuring small, time-resolvable changes in membrane capacitance (ΔC_m) from single chromaffin cells using a modified Axopatch 200A amplifier and phase-tracking software as per Giovannucci et al. (24). Recordings were performed using borosilicate pipettes constructed out of 1.5 mm outer diameter capillary glass tubing containing a filament with flame polished ends. The pipette was coated with a Sylgard elastomer and fire polished to resistances of 1.5–3 MΩ. The standard intracellular recording solution consisted of 20 mM CsCl, 120 mM $MeSO_4$, 120 mM CsOH, 10 mM HEPES, 1 mM $MgCl_2$, 0.1 mM EGTA, 1 mM HEDTA (pH 7.2) with CsOH. A concentrated stock solution of amphotericin B (30 μg/μL) was made fresh for each experiment and added before recording. External solution consisted

TABLE 1 Sequences of qPCR primers

Gene	Forward primer	Reverse primer
β Actin	CCACCATGTACCCAGGCATT	CAGCTCAGTAACAGTCCGCC
PKCα	TTGTCCAAGGAAGCCGTCTC	CCTTTGCCACACACTTTGGG
PKCβ	GAACCACAAATTCACCGCCC	GCAGCAGACTTGACACTGGA
PKCγ	TTACAATGTACCGGTGGCCG	CATCGCTTGGAGTCCGTAGG
PKCδ	AACTGGTCCCTCCTGGAGAA	GCTTCCTGGTCCATAGAGTCG
PKCη	TACCTGACGGTGAGCGTAGA	AGATCCACCCAGCCCTCGAA
PKCΘ	GCTTGTCAAAGAGTATGTGGAATCA	TCGGCCTTGAGGTTTCAGC
PKCε	CAGGGTATCTGGGGAATGGC	GTCCATAATGAGAGGCAGGGA
PKCμ	TGTGTTTGGTGTGCTCCCAT	CCCCCACCTACTGTGCATTT

of 137 mM TEA-Cl, 10 mM $CaCl_2$, 10 mM HEPES, 2 mM $MgCl_2$, 19 mM glucose (pH 7.2), with Tris base.

To determine the relationship between Ca^{2+} entry and secretory output, a train of 12-step depolarizations with a duration of 50 ms from a holding potential of −90–0 mV at a 0.5 s interval were applied to evoke I_{Ca} and ΔC_m. Evoked Ca^{2+} currents were time integrated, and charge (pC) was related to the corresponding increases in membrane capacitance (C_m). The measured increases in Ca^{2+} entry and C_m were plotted as cumulative charge (ΣpC) versus cumulative change in membrane capacitance (ΣC_m). The slope of this relationship was fitted by a linear function and estimates of the Ca^{2+} dependence of secretory activity were obtained as per Giovannucci et al. (24,25) and Yang et al. (26). The immediately releasable pool (IRP) of vesicles was estimated by the first step change in capacitance (fF_1) normalized to Ca^{2+} charge entry (pC_1). The RRP of vesicles was estimated from the last cumulative step change in capacitance (fF_{max}) normalized to the total Ca^{2+} charge (ΣpC) after the applied pulse train as per Giovannucci et al. (24).

To measure PACAP-mediated effects on activity-dependent chromaffin cell exocytosis, a standard pulse train was applied to cells treated for 1 min by local bath application with PACAP (0.5 μM) after application of a control pulse train. Cells were maintained in PACAP during the second pulse train. PACAP-mediated effects on exocytosis Ca^{2+} sensitivity, IRP, and RRP size were determined as described above. To determine whether the PACAP-mediated effect on the Ca^{2+}-exocytosis relationship was dependent on PKC activity, experiments were carried out in either the presence or absence of the PKC inhibitor NPC 15437 (10 μM). Cells were treated with NPC 15437 for 5 min before application of a control pulse train, with NPC 15437 maintained during application of a subsequent train after treatment with PACAP.

Statistical analysis

GraphPad Prism was used for all statistical analysis. The distribution of values for a particular data set were first assessed for normality with a Shapiro-Wilk test. Differences in means of normally distributed data were subsequently compared using a Student's *t*-test with Welch's correction. A Mann-Whitney or Wilcoxon matched-pairs signed-rank test was used to compare data sets whose individual values were not normally distributed, as noted. For multiple comparisons, either a one-way ANOVA (normally distributed data) with Tukey's test or Kruskal-Wallis (postnormally distributed data) with Dunn's test was used to compare data sets. Plots and graphs report means ± standard deviation (SD) or mean ± SE as indicated. Within figures, ns = $p > 0.05$; *$p < 0.05$, **$p < 0.01$, ***$p < 0.001$, and ****$p < 0.0001$.

RESULTS

PACAP-stimulated DAG production is PLCε dependent

DAG is a lipid second messenger produced by the hydrolysis of phosphatidylinositol PIP_2 by PLC (27–29) (Fig. 1 *A*). DAG and PKC are known to regulate various aspects of exocytosis, including vesicle priming and fusion, in chromaffin cells (22,26,30–32). Hence, our first goal was to show PACAP stimulation causes DAG production downstream of PLCε. To this end, we harvested chromaffin cells from WT and PLCε knockout (KO) mice and monitored DAG production optically using a fluorescent DAG reporter (33). The fluorescent intensity of the sensor increases upon binding to DAG, allowing real-time monitoring of DAG production by TIRF microscopy.

Representative TIRF images of a WT and a PLCε KO cell expressing the DAG sensor are shown in Fig. 1 *B*. The image series in the upper panels (WT cell) indicate that DAG sensor fluorescence increased markedly upon 500 nM PACAP stimulation, consistent with robust DAG production. In contrast, there was little change in DAG sensor fluorescence in the KO cell. The corresponding percentage change in fluorescence ($\%\Delta F/F_0$) over time records for these, and other cells, were averaged and are displayed in Fig. 1 *C*. The maximum change in DAG sensor fluorescence in response to PACAP was also calculated. Individual points, representing values from separate cells, are provided in Fig. 1 *D*. Taken together, the data demonstrate that PACAP-stimulated DAG production requires PLCε expression.

The PKC inhibitor NPC 15437 disrupts PACAP-evoked Ca^{2+} transients and secretion

PKC is a known effector of DAG and, as referenced earlier, has been reported to play multiple roles in chromaffin cell exocytosis. To disrupt the activation of PKC by DAG, we incubated chromaffin cells with NPC 15437 (abbreviated hereafter as NPC)—a specific inhibitor of DAG binding to the C1 domain of PKC—before PACAP stimulation (34).

Ca^{2+} signals evoked by 500 nM PACAP stimulation were robust and fluctuated with a variable amplitude and frequency (Fig. 2 *A*). In contrast, when cells were stimulated by PACAP in the presence of 1 μM NPC, the Ca^{2+} signals were attenuated, exhibiting fewer, smaller-amplitude spikes (Fig. 2, *A* and *B*). Representative images illustrate the diminished fluorescent responses when NPC was included in the bath (Fig. 2 *B*). To quantify the effect of PKC inhibition on the fluorescent responses, the maximum change in

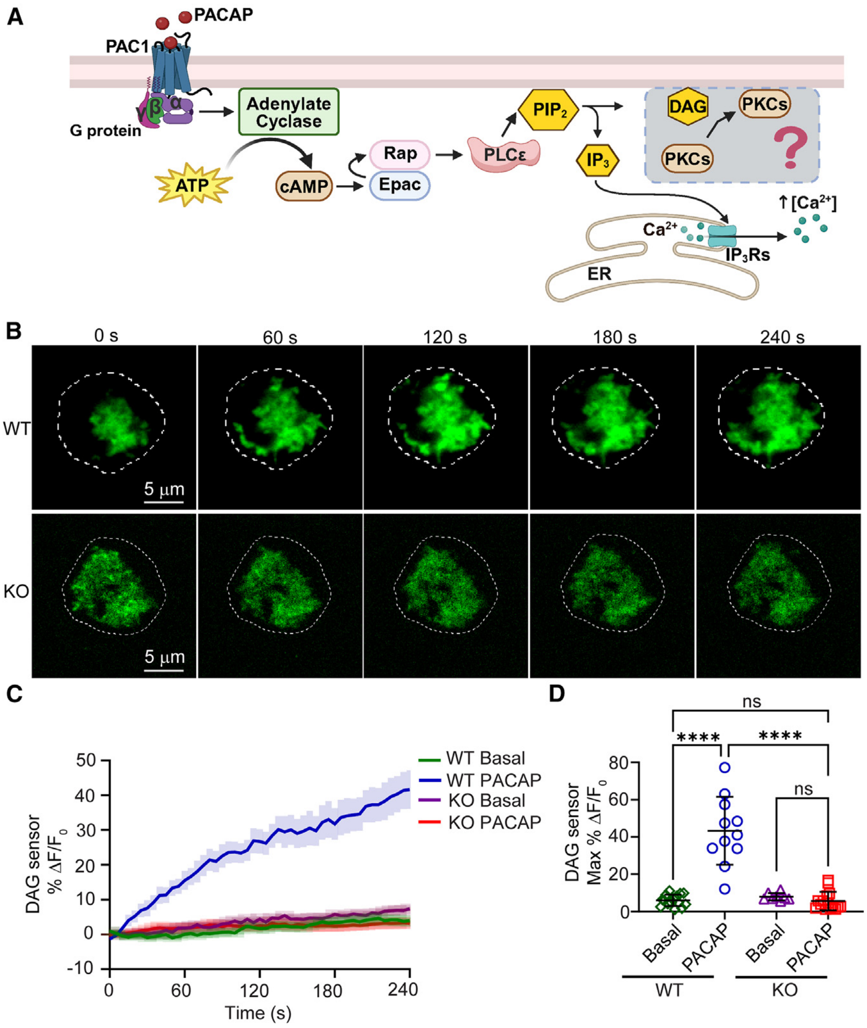

FIGURE 1 PACAP-stimulated DAG production is impaired in PLCε KO cells. (*A*) A PACAP-triggered signaling cascade regulates Ca^{2+} transients in chromaffin cells. PACAP binds to its high-affinity receptor, PAC1, activating $G\alpha_s$. $G\alpha_s$ stimulates adenylate cyclase, leading to cAMP production. Elevated cAMP activates Epac and, subsequently, PLCε. PLCε hydrolyzes PIP_2 into two key signaling molecules: IP_3 and DAG. IP_3 binds to its receptors on the endoplasmic reticulum, triggering Ca^{2+} release into the cytosol. This study investigates the consequences of the DAG signaling axis (*boxed*). (*B*) Representative images obtained by TIRF imaging of WT and PLCε KO cells expressing a DAG sensor during stimulation (begins at time 0) with 500 nM PACAP. The images show changes in fluorescence intensity over time, indicating DAG production. Dotted lines indicate the cell boundaries based on bright-field images. Scale bars, 5 μm. (*C*) The percentage change in fluorescence (%$\Delta F/F_0$) versus time record of the DAG sensor in WT and PLCε KO cells under basal conditions (physiological saline solution [PSS]) and during PACAP stimulation. The graph depicts the time course of DAG production after PACAP stimulation, with bold lines representing the mean response and shaded areas representing the standard error of the mean. Data were collected from two independent experiments. Sample sizes are $n = 15$ (WT basal), $n = 11$ (WT PACAP), $n = 9$ (KO basal), and $n = 15$ (KO PACAP). (*D*) Scatterplots showing the individual maximum percent change in DAG sensor fluorescence in response to PACAP in both WT and PLCε KO cells, derived from the data shown in (*C*). Data are presented as mean ± SD. Statistical significance: $^{****}p < 0.0001$; ns, not significant. Statistical significance was assessed using one-way ANOVA with Tukey's multiple comparisons test. Not all comparisons are shown for clarity.

Lck-GCaMP5G fluorescence (%$\Delta F/F_0$) and the total spike area (i.e., area under the curve) were measured (Fig. 2, *D* and *E*). The data show the strong dependence of PACAP-stimulated Ca^{2+} responses on PKC activity, with NPC reducing the amplitude and integrated intensity of the fluorescent signals.

Next, we examined the impact of PKC inhibition on PACAP-stimulated exocytosis using NPY-pHluorin, a pH-sensitive fluorescent reporter of secretory granule fusion (35,36) (Fig. 2, *C* and *F*). PACAP (500 nM) stimulation triggered numerous fusion events, visualized as sudden increases in fluorescence intensity at discrete locations on the cell footprint (indicated by *arrows* in Fig. 2 *C*). Quantification of these events revealed that PKC inhibition by NPC caused a significant, dose-dependent decrease in the number of fusion events per unit area (Fig. 2 *F*). This reduction in exocytosis correlated well with the observed attenuation of Ca^{2+} signals. These results suggest that the PACAP-evoked Ca^{2+} transients and exocytosis in chromaffin cells are dependent on DAG-stimulated PKC activity.

PKC inhibition eliminates PACAP-mediated increases in RRP size and Ca^{2+} sensitivity of fusion

To further our understanding of the role of PKC in PACAP-evoked exocytosis, we used time-resolved membrane capacitance methods to assay secretory activity in single chromaffin cells under perforated patch-clamp configuration. To determine whether PACAP might regulate activity-dependent secretion, we measured cell capacitance changes (ΔCm) in response to a series of step depolarizations before and after local bath application of PACAP. As shown in Fig. 3 *A*, Ca^{2+} entry during each step depolarization was quantified by time integration of the Ca^{2+} current, expressed as charge (pC), and correlated to each stepwise increase in cell capacitance (fF). The relationship between cumulative charge (pC) was plotted against the cumulative changes in capacitance (fF) to define an input/output transfer function as shown in Fig. 3 *B*. As shown in Fig. 3, *C* and *E*, PACAP treatment significantly increased the slope of the

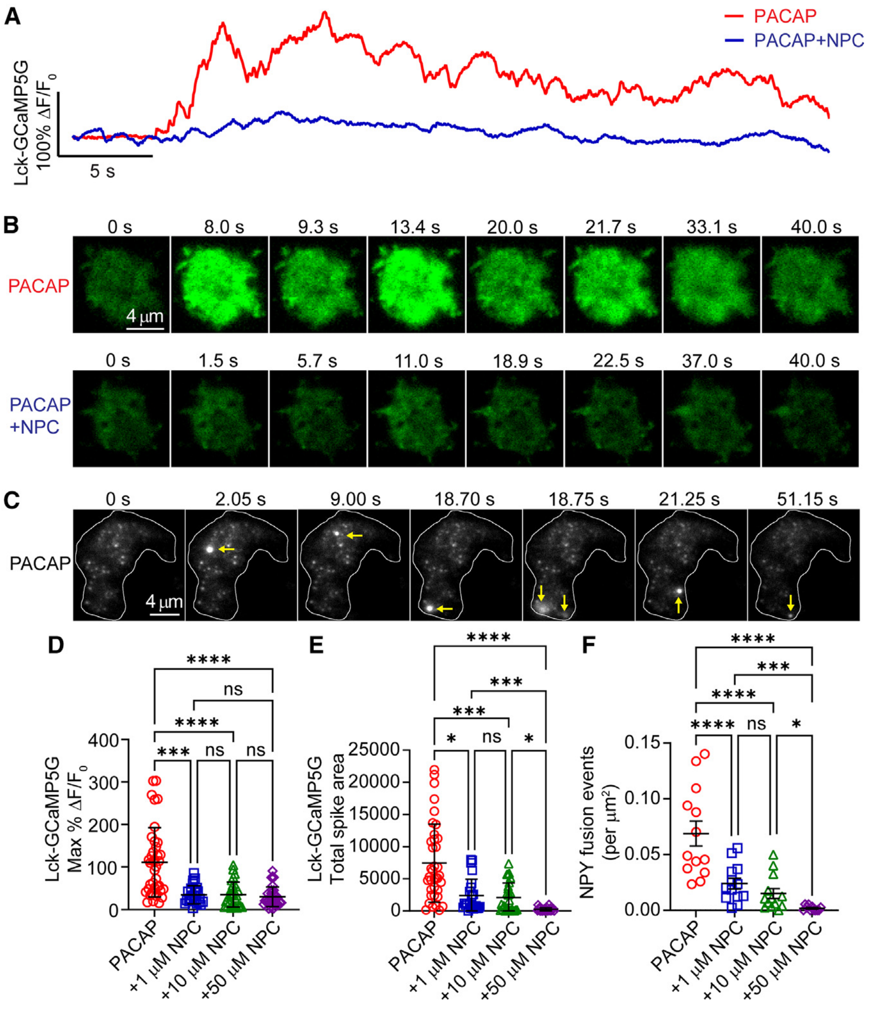

FIGURE 2 PACAP-stimulated Ca^{2+} signals and exocytosis are inhibited by NPC 15437 in a dose-dependent manner. (*A*) Representative % $\Delta F/F_0$ versus time trace for chromaffin cells expressing Lck-GCaMP5G and stimulated with either 500 nM PACAP alone or 500 nM PACAP + 1 μM NPC 15437 (NPC) for 45 s. Experiments were performed on a TIRF microscope. (*B*) Time series images of cells expressing Lck-GCaMP5G during stimulation with 500 nM PACAP or 500 nM PACAP + 1 μM NPC, in the PACAP + NPC group, NPC was also applied to the bath at least 1 min before stimulation. Scale bars, 4 μm. (*C*) Representative images of a cell expressing NPY-pHluorin stimulated with 500 nM PACAP. The outline of the cell footprint is indicated by the white lines. Arrows show the location of individual NPY fusion events in the time series. (*D*) Maximum % $\Delta F/F_0$ for chromaffin cells stimulated with 500 nM PACAP alone or with three different concentrations of NPC (1, 10, and 50 μM). Sample sizes are n = 38 (PACAP), n = 24 (+1 μM NPC), n = 28 (+10 μM NPC), and n = 31 (+50 μM NPC). (*E*) PACAP-stimulated Ca^{2+} spike area was reduced by NPC in a dose-dependent manner. Chromaffin cells stimulated with 500 nM PACAP alone or with three different concentrations of NPC (1, 10, and 50 μM). Sample sizes are n = 37 (PACAP), n = 24 (+1 μM NPC), n = 28 (+10 μM NPC), and n = 27 (+50 μM NPC). (*F*) PACAP-stimulated exocytosis was reduced by NPC in a dose-dependent manner. Chromaffin cells stimulated with 500 nM PACAP alone or with three different concentrations of NPC (1, 10, and 50 μM) for 45 s. Sample sizes are n = 13 (PACAP), n = 13 (+1 μM NPC), n = 13 (+10 μM NPC), and n = 11 (+50 μM NPC). In all PACAP +NPC groups, NPC was also applied to the bath at least 1 min earlier. Data were collected from 3 independent experiments and are presented as mean ± SD. Statistical significance was assessed using one-way ANOVA with Kruskal-Wallis test. Statistical significance: *$p < 0.05$, ***$p < 0.001$, ****$p < 0.0001$; ns, not significant.

pC/ΔCm relationship and enhanced the apparent size of the RRP of granules compared with controls ($n = 18$; $p < 0.05$). Since similar enhancements of exocytosis in mouse chromaffin cells after PMA treatment—and activation of PKC—have been reported by others and in our supporting material (Fig. S1), we tested whether the effects of PACAP on activity-dependent chromaffin cell exocytosis was dependent on PKC (26,32). To prevent PKC activation, we again used the NPC 15437 compound. Following treatment with the inhibitor, PACAP-induced increases in the input/output relationship and RRP size were largely abolished and both parameters were restored to levels comparable with the control condition. These data suggest that PKC activation is crucial for the PACAP-induced enhancement of activity-evoked exocytosis (Fig. 3 *C*). Interestingly, neither PACAP treatment nor PKC inhibition had any significant effect on the IRP of granules (Fig. 3 *D*).

We next determined whether the PACAP-mediated enhancement of activity-dependent exocytotic activity might be explained by an increase in the evoked Ca^{2+} currents. To do so, I-V relationships were generated before and after treatment with PACAP using a step depolarization paradigm. These data indicated that PACAP treatment induced no significant enhancement of peak Ca^{2+} current amplitudes (Fig. S1 *E*). The lack of a change in Ca^{2+} current suggested that the PACAP-mediated enhancements of chromaffin cell secretory activity are likely through an enhancement in Ca^{2+} sensitivity for fusion and/or the number of granules in the RRP. To investigate potential effects of PACAP on Ca^{2+} channel activation, we measured the voltage dependence of Ca^{2+} currents before and after incubation of PACAP in chromaffin cells using a voltage-ramp stimulation (Fig. S1 *F*). Compared with control ramps, PACAP treatment enhanced Ca^{2+} channel activation at more negative voltages (Fig. S1 *F*). When the ramp duration values were transformed to voltage values, there appeared to be a modest enhancement of currents at −48, −32, and −16 mV in PACAP-treated cells. These results suggest

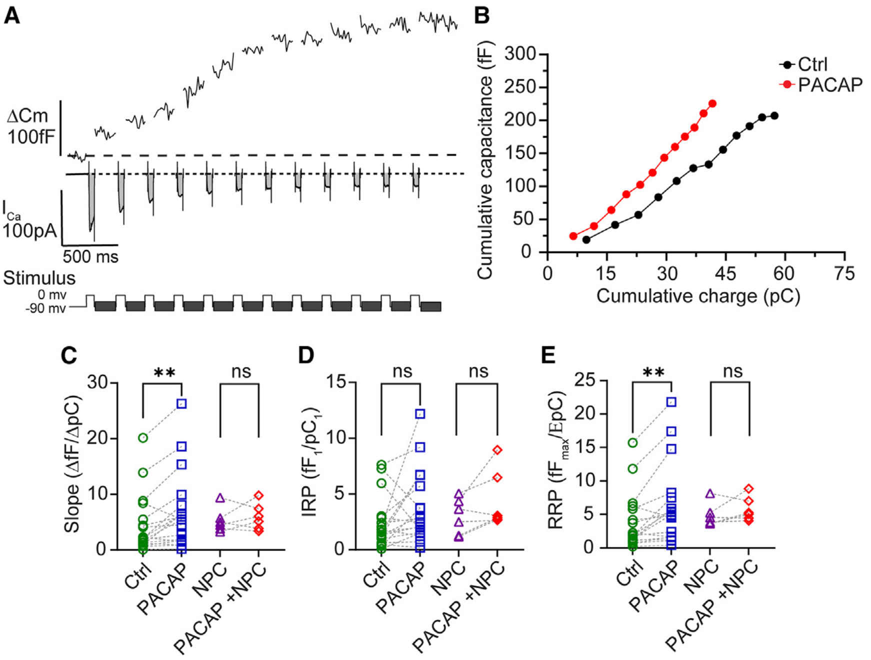

FIGURE 3 PACAP-mediated enhancement of activity-dependent exocytosis requires PKC signaling. (*A*) An example trace of step depolarization-evoked changes in whole-cell capacitance in a chromaffin cell. Membrane capacitance was measured before and after each step depolarization and the corresponding Ca^{2+} currents (I_{ca}) were time integrated to estimate the Ca^{2+} charge entry. Cells were held at -90 mV and currents were evoked by 50 ms step depolarizations to 0 mV. (*B*) Cumulative changes in charge (pC) were plotted against cumulative membrane capacitance changes (fF) to assess the input-output relationship. (*C*) A comparison of the pC/ΔC_m relationship between cells pretreated with or without the PKC inhibitor NPC (10 μM) for 5 min, and subsequently treated with PACAP (500 nM). PACAP was bath applied for 1 min after a control train and maintained during the second pulse train ($n = 18$). NPC was maintained during the treatment train ($n = 6$). Ca^{2+} sensitivity of exocytosis was enhanced by PACAP. The PACAP-mediated enhancement was abolished when the cells were pretreated with NPC ($p = 0.5$). (*D*) There was no change in IRP size of PACAP-treated cells either in the absence ($p = 0.06$) or presence of NPC ($p = 0.09$). (*E*) There was a significant increase in the RRP measured for PACAP-treated cells that was eliminated by NPC treatment ($p = 0.2$). Statistical significance: $**p < 0.01$; ns, not significant.

that Ca^{2+} channels activated at lower potentials may be particularly important for the PACAP-dependent effects (34,37). Consistent with this idea, PKC inhibition abrogated the PACAP-mediated, leftward shift in Ca^{2+} channel activation (Fig. S1 *G*).

PACAP causes PKC C1b domain translocation in WT cells but not in PLCε KO cells

PKC is characterized by its conserved structure, which includes a regulatory domain and a catalytic domain (38). The regulatory domain contains several subdomains, including C1a and C1b. Binding of DAG to the PKC C1b domain underlies its translocation to the plasma membrane (23). This event is a critical step in the PKC signaling pathway (38). To determine whether PACAP stimulation can cause C1b redistribution in chromaffin cells, we overexpressed the PKCβ C1b domain as an EGFP fusion protein. As shown in Fig. 4 *A*, WT cells expressing C1b-EGFP exhibited a marked increase in fluorescence at the plasma membrane after stimulation with both 1 and 5 μM PACAP. In contrast, little to no change in near-membrane fluorescence of EGFP was detected in PLCε KO cells stimulated by PACAP, suggesting that C1b translocation was impaired. Fig. 4 *B* shows the percentage change in fluorescence (%$\Delta F/F_0$) over time for C1b-EGFP in both WT and PLCε KO cells during basal conditions and after PACAP stimulation. WT cells exhibited a significant and sustained increase in C1b-EGFP fluorescence upon PACAP stimulation, with higher concentrations of PACAP (5 μM) eliciting a more robust response compared with lower concentrations (1 μM) (Fig. 4 *C*). There was little change in fluorescence detected in PLCε KO cells, regardless of PACAP concentration (Fig. 4 *D*). This observation supports a critical role for PLCε in regulating PACAP-stimulated PKC activation.

We next confirmed that antagonizing the DAG-PKC interaction would also inhibit PACAP-stimulated PKC translocation. Cells overexpressing the PKC C1b domain construct were exposed to an initial basal wash or were preincubated with NPC (Fig. S2 *A*). Cells were then stimulated with PACAP or PACAP + NPC. As shown in Fig. S2, little or no increase in fluorescence intensity of C1b-EGFP within the evanescent field was detected when stimulation was performed in the presence of the inhibitor. Thus, the data support a model where the activity of PLCε, DAG, and PKC are all important in regulating downstream events associated with PACAP stimulation.

Identification of PKC subtypes activated by PACAP stimulation

Having demonstrated that DAG-stimulated PKC activity has an important role in the PACAP secretory response, we next sought to identify which specific isoforms might be involved. To do so, we first assessed the expression of eight PKC isoforms belonging to the "conventional" (PKCα, PKCβ, PKCγ), "novel" (PKCδ, PKCε PKCη, PKCθ), and "atypical" (PKCμ) subfamilies (38,39). Five isoforms (PKCα, PKCβ, PKCδ, PKCε, and PKCμ) were reliably detected in mouse chromaffin cells (Fig. 5 *A*). Of these,

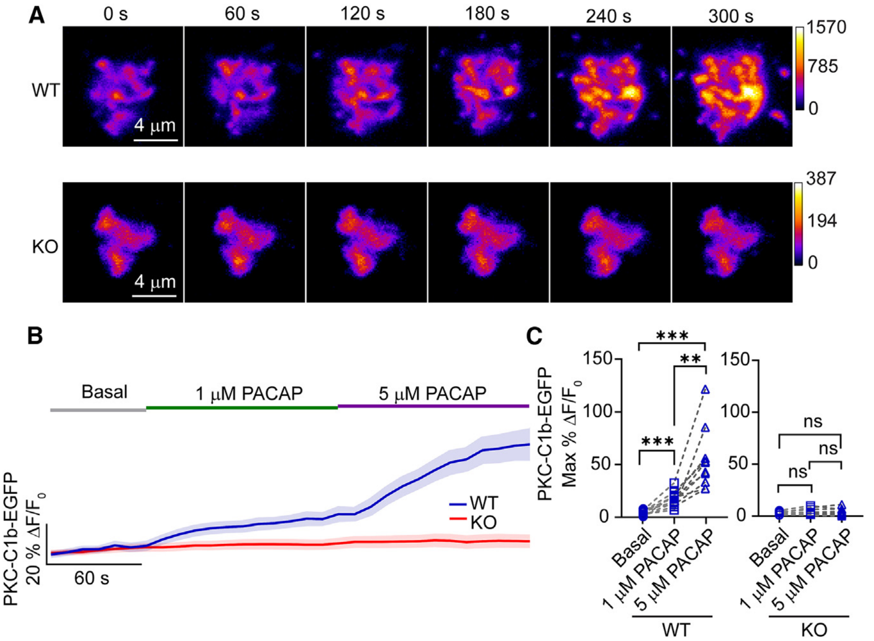

FIGURE 4 PACAP-stimulated PKC C1b-EGFP translocation in WT and PLCε KO chromaffin cells. (*A*) Representative time-lapse images of WT and PLCε KO cells expressing C1b-EGFP before and during stimulation with 1 and 5 μM PACAP. Color is transformed to encode increases in intensity, as indicated. Images of WT cells show an increase in EGFP fluorescence within the TIRF evanescent field, consistent with C1b domain translocation to the plasma membrane; KO cells exhibit minimal changes. Scale bars, 4 *a*m. (*B*) The percentage change in fluorescence (%$\Delta F/F_0$) over time for C1b-EGFP in both WT and PLCε KO cells during basal conditions and PACAP stimulations. The graph illustrates the time course of C1b recruitment to the plasma membrane, with bold lines representing the mean response and shaded areas representing the mean ± SE. (*C*) Scatterplots showing the individual maximum percentage change in C1b-EGFP fluorescence in response to PACAP in both WT and PLCε KO cells. Data were collected from 3 independent experiments. Statistical significance was assessed using one-way ANOVA with Tukey's multiple comparisons test. WT cells, $n = 10$, KO cells, $n = 12$. Statistical significance: $^{**}p < 0.01$, $^{***}p < 0.001$; ns, not significant.

transcripts for PKCβ, PKCε, and PKCμ were the most abundantly expressed. Protein expression of these PKCs was substantiated by western blotting (Fig. 5 *B*).

Next, we investigated the functional consequences of "knocking down" PKCβ, PKCε, and PKCμ expression on PACAP-evoked Ca^{2+} transients in chromaffin cells. Cells were transfected with shRNAs targeting PKCβ, PKCε, and PKCμ, or with scrambled shRNA controls. Transfected cells were identified on the basis of their red fluorescence (PKCμ and PKCε shRNAs) or green fluorescence (PKCβ shRNA), necessitating the use of different Ca^{2+} indicators as shown (Fig. 6). Cells expressing shRNAs for PKCβ and PKCε exhibited significantly reduced PACAP-evoked Ca^{2+} signals compared with the scrambled shRNA controls ($p < 0.0001$) (Fig. 6, *A–F*). In contrast, expression of the PKCμ shRNA did not significantly affect the Ca^{2+} responses to PACAP stimulation compared with its scrambled control (Fig. 6, *G–I*). To assess knockdown efficiency, chromaffin cells expressing the various shRNAs were selected with 100 μg/mL puromycin for 3 days to enrich for transfected cells. Quantitative PCR was then used to measure changes in mRNA expression levels. As shown in Fig. S3, all three shRNAs (PKCβ, PKCε, and PKCμ) significantly reduced mRNA expression by more than 70% compared with scrambled shRNA controls.

Knockdown of both PKCβ and PKCε expression significantly reduced Ca^{2+} responses to PACAP stimulation. Thus, we predicted that their knockdown would also impact the secretory response. To test this idea, chromaffin cells expressing either the scrambled, PKCβ, or PKCε KD shRNAs

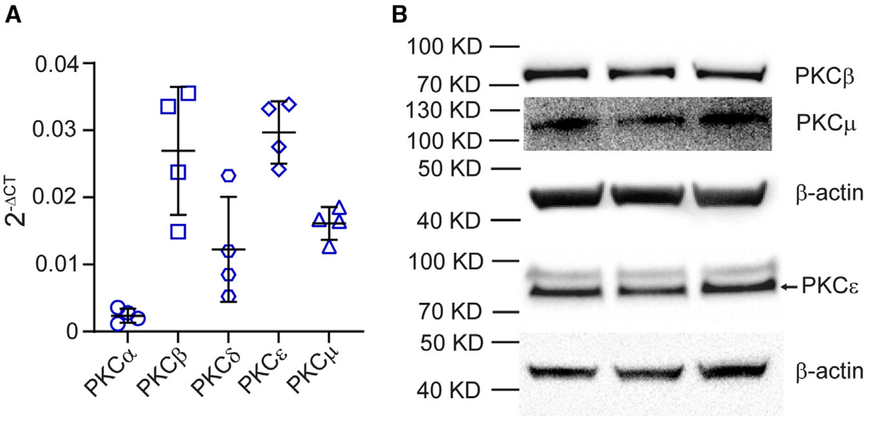

FIGURE 5 Identification of PKC subtypes in chromaffin cells. (*A*) qPCR analysis of PKC isoform expression in mouse chromaffin cells. The expression levels of eight PKC isoforms (PKCα, PKCβ, PKCγ, PKCδ, PKCε, PKCη, PKCθ, and PKCμ) were measured. PKCα, PKCβ, PKCδ, PKCε, and PKCμ transcripts were detected; PKCβ, PKCδ, PKCε, and PKCμ transcripts were the most abundant. Data are presented as mean ± SD from two independent experiments, total using four different samples. (*B*) Western blot analysis confirms the expression of PKCβ, PKCε, and PKCμ in adrenal medulla. Samples were obtained from three WT mice, with each lane representing an individual mouse. β-Actin was used as a loading control.

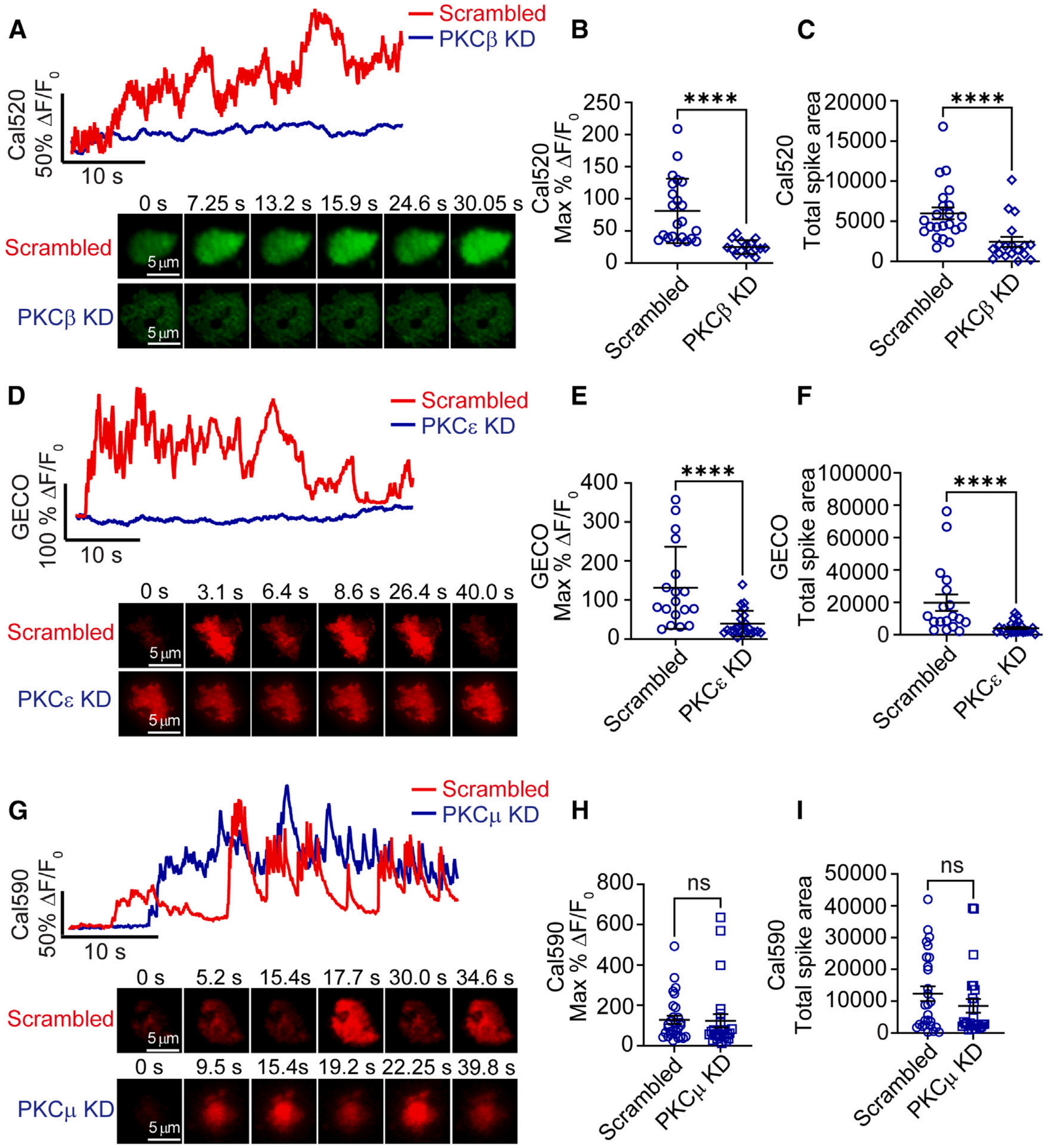

FIGURE 6 PKCβ and PKCε, but not PKCμ, knockdown attenuated PACAP-evoked Ca^{2+} transients in chromaffin cells. (*A*) Representative trace of $\%\Delta F/F_0$ versus time for PKCβ knockdown and scrambled shRNA-transfected cells loaded with Cal520 and stimulated with 500 nM PACAP. The bottom images illustrate the signal changes at different time points in the same cell as shown in the intensity versus time record. (*B*) Knockdown of PKCβ significantly reduced the maximum amplitude of PACAP-induced Ca^{2+} transients. PKCβ knockdown cells exhibited an approximately 70% reduction in the maximum $\%\Delta F/F_0$. Data are presented as mean ± SD. (*C*) The total spike area of Ca^{2+} transients was also significantly decreased in PKCβ knockdown cells compared with scrambled control cells. Chromaffin cells were loaded with 1 μM Cal520 for 30 min after transfection with the shRNA plasmid for at least 48 h. Knockdown cells were identified by RFP expression from the shRNA vector. Data are from two independent experiments ($n = 22$ scrambled, $n = 16$ PKCβ KD). (*D*) Representative trace of $\%\Delta F/F_0$ versus time for PKCε knockdown and scrambled shRNA-transfected cells stimulated with PACAP. The bottom images show fluorescence signal changes at different time points in the same cell as shown in the trace. (*E*) Knockdown of PKCε using shRNA significantly reduced the maximum amplitude of PACAP-induced Ca^{2+} transients. PKCε knockdown cells showed an approximately 70% reduction in the maximum $\%\Delta F/F_0$ compared with scrambled shRNA control cells. Data are presented as mean ± SD. (*F*) The total spike area of Ca^{2+} transients was also significantly decreased in PKCε knockdown cells compared with scrambled control cells. Chromaffin cells were cotransduced with red GECO and the shRNA plasmid. Knockdown cells were identified by GFP expression from the shRNA vector. Data are from two independent experiments ($n = 18$ scrambled, $n = 24$ PKCε KD). (*G*) Representative trace of $\%\Delta F/F_0$ versus time for PKCμ knockdown and scrambled shRNA-transfected cells stimulated with PACAP. The bottom images illustrate fluorescence signal changes at different time points in the same cell as shown in the trace. (*H*) Knockdown of PKCμ using shRNA did not significantly

(legend continued on next page)

(identified here by their red fluorescence) were incubated with FFN511 to label secretory granules (40). FFN511 is a fluorescent analog of dopamine and is reliably taken up into chromaffin cell granules, ostensibly via endogenous VMAT-dependent transport (40). Cells loaded with FFN511 and expressing the shRNAs were subsequently stimulated with 500 nM PACAP for 45 s while secretion was monitored in TIRF (Fig. 7 *A*). Knockdown of PKCβ and PKCε expression significantly reduced the number of fusion events per μm^2 compared with scrambled controls, as shown ($p < 0.0001$) (Fig. 7, *B* and *C*).

DISCUSSION

Investigations on the effects of PACAP on chromaffin cells agree on key points: 1) PACAP causes a long-lasting secretory response and 2) the PACAP secretory response is triggered by a persistent increase in intracellular Ca^{2+}. The increase in intracellular Ca^{2+} is a consequence of Ca^{2+} influx through plasma membrane channels and Ca^{2+} release from the ER (i.e., Ca^{2+}-induced Ca^{2+} release) (12,17,41–43). These Ca^{2+} changes depend on signaling events downstream of PLCε (12). The precise role of those downstream signaling events are not yet entirely apparent, but experiments performed in this study provide some important clues.

Delineating a role for a PLCε-DAG-PKC signaling module in exocytosis

A conserved function of PLCε, established from experiments in a variety of systems, is to hydrolyze PIP_2 to generate IP3 and DAG (20) (Fig. 8). In chromaffin cells stimulated by PACAP, IP3 production gates the release of Ca^{2+} from the ER. When this process is disrupted by either inhibiting IP3 receptor activity with Xestospongin C or knocking down IP3R1 expression, Ca^{2+} signals evoked by PACAP are significantly reduced (12). Our results now also define important roles for DAG-mediated signaling in chromaffin cells. Here, we showed that PACAP stimulation of chromaffin cells generated DAG in a manner that was dependent on PLCε activity (Figs. 1 and 8). DAG is itself a known activator of PKC, with a hallmark of PKC activation being its translocation to the membrane (38,44). We monitored PKC translocation in TIRF using a PKCβ C1b domain fused to EGFP (Fig. 4). Because PKC translocation was disrupted by NPC 15437, and in the PLCε KO, this process clearly required intact DAG signaling. The Ca^{2+} signals evoked by PACAP, as well as exocytosis, were attenuated by PKC inhibition. These findings indicate that PKC signaling cascades, activated by DAG through PLCε, regulate the secretory response to PACAP stimulation.

It has been known for some time that application of phorbol esters potentiates the secretory response in chromaffin cells, and that this effect is blocked by PKC inhibition (45,46). What is especially novel here is the discovery that this potentiation occurs downstream of a regulated pathway for secretion stimulated by PACAP (26,32,47). Indeed, we found that PACAP not only caused secretion but sensitized chromaffin cells to further stimulation (Figs. 3 and S1). These changes, perceptible even after a 1 min exposure to PACAP, were inhibited when PKC activation was prevented. There are multiple molecular loci on which PKCs might act to modulate the secretory response. It is well known, for example, that PKC signaling can influence the activation of a variety of voltage-gated channels, of which chromaffin cells express many (34,48). This topic has been extensively reviewed elsewhere (48). However, PKCs may also act directly on elements of the release apparatus in addition to channels (49). Compelling evidence of roles for Munc18-1, SNAP-25, and/or synaptotagmin-1 phosphorylation (by PKA or PKC) in augmenting granule pool size/refilling rates have been published (26,30,50–53). The molecular basis for these effects are not clear, but may involve changes in binding efficacy of the respective proteins to other SNAREs or SM proteins (49).

Multiple PKC isoforms are expressed in chromaffin cells (54–57). Here, we provide evidence that knockdown of either PKCβ or PKCε caused a reduction in PACAP-stimulated Ca^{2+} transients. The observation that PKCε is involved in this pathway should perhaps not be surprising. Indeed, it was shown previously that PKCε is important for potentiating secretion triggered by repeated acetylcholine stimulation (56). PKCε belongs to a class of "novel" PKCs, whose activation depends solely on DAG and not Ca^{2+} (38). Thus, DAG generated by PLCε-stimulated PIP_2 hydrolysis alone is sufficient to activate PKCε. We also found that knockdown of PKCβ reduced PACAP-stimulated Ca^{2+} transients and exocytosis. Unlike PKCε, however, PKCβ requires both DAG and Ca^{2+} for its activation (38). Thus, these PKCs may be activated in sequence, with a PKCε-dependent mechanism first gating an increase in cytosolic Ca^{2+}, which then permits the activation of PKCβ.

A model for PACAP-stimulated secretion in the adrenal medulla

PACAP has an established role in regulating chromaffin cell secretion. However, the mechanisms by which it

affect the maximum amplitude of PACAP-induced Ca^{2+} transients. Data are presented as mean ± SD. (*I*) The total spike area of Ca^{2+} signals was not significantly reduced in PKCμ knockdown cells compared with scrambled control cells. Chromaffin cells were loaded with 1 μM Cal590 for 30 min after transfection with the shRNA plasmid for at least 48 h. Knockdown cells were identified by GFP expression from the shRNA plasmid. Data are from two independent experiments ($n = 29$ scrambled, $n = 25$ PKCμ KD). Statistical significance was determined using a two-tailed unpaired *t*-test. ****$p < 0.0001$; ns, not significant.

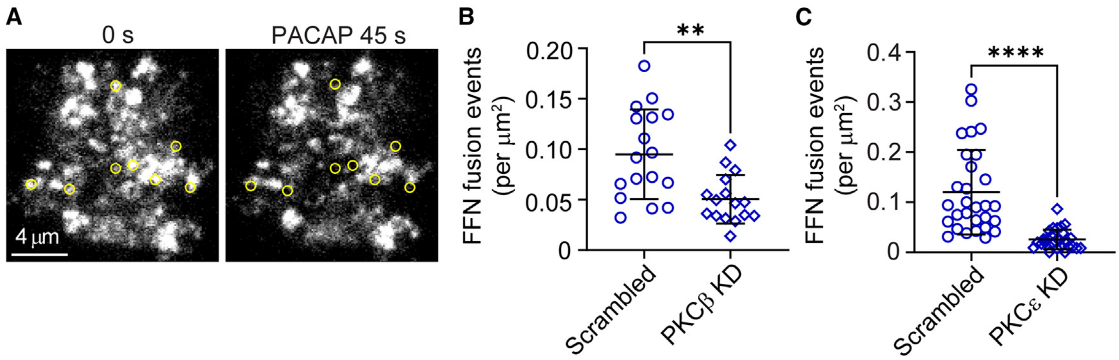

FIGURE 7 Knockdown of PKCβ and PKCε significantly disrupted PACAP-stimulated exocytosis. (*A*) Representative images of a chromaffin cell with granules loaded with FFN511 (abbreviated FFN) before and after stimulation with PACAP for 45 s. Yellow circles indicate the locations where fusion events occurred. (*B*) Knockdown of PKCβ significantly reduced the number of FFN fusion events per μm^2 compared with scrambled shRNA control cells ($p = 0.001$). Data were collected from two independent experiments ($n = 17$ for scrambled, $n = 16$ for PKCβ KD). (*C*) Knockdown of PKCε significantly reduced the number of FFN fusion events per μm^2 compared with scrambled shRNA control cells ($p < 0.0001$). Data were collected from two independent experiments ($n = 29$ for scrambled, $n = 27$ for PKCε KD). Chromaffin cells were loaded with 20 μM FFN511 for 30 min after transfection with the shRNA plasmid. Knockdown cells were identified by RFP expression and were stimulated by 500 nM PACAP for 45 s. Data are presented as mean ± SD. Statistical significance was determined using a two-tailed unpaired *t*-test (**$p < 0.01$, ****$p < 0.0001$).

does so remain opaque. In this study, we have delineated novel functions for PKC, downstream of PLCε, in the PACAP secretory response. A goal of future work will be to define the loci at which PKCs act to facilitate fusion. Our results also suggest that PACAP not only causes secretion but lowers the threshold for additional secretion whether elicited by PACAP or other stimuli. Such a model is not without precedent. It was shown previously at autonomic ganglia that PACAP has both short- and longer-term effects on nicotinic-receptor driven cellular excitability—effects that arise from activation of cAMP-dependent signaling cascades and gene transcription, respectively (58). In chromaffin cells, even short-term (∼1 min) application of PACAP augments cellular excitability (i.e., by increasing Ca^{2+} currents at negative potentials) and the secretory response (i.e., by sensitizing granules to Ca^{2+} signals and increasing the size of the RRP).

In summary, these novel findings show that PACAP both directly causes exocytosis and sensitizes exocytosis to further stimulation. The "feedforward" effects of PACAP on the secretory response may become especially important when the demand for adrenomedullary hormones is elevated during the sympathetic stress response.

ACKNOWLEDGMENTS

This research was supported by NIH grant R01 NS122534 awarded to A.A. B.L.C. is supported by NIH fellowship T32 GM144873.

AUTHOR CONTRIBUTIONS

A.A., D.R.G., X.C., and N.A.B. designed the experiments. X.C., N.A.B., and B.L.C. performed the experiments. X.C. and N.A.B. analyzed the data. X.C. and A.A. wrote the manuscript.

DECLARATION OF INTERESTS

The authors declare no competing interests.

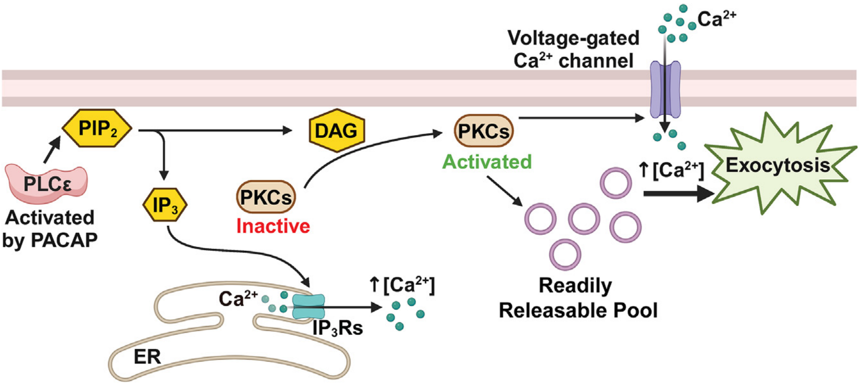

FIGURE 8 Roles for PKC signaling in the PACAP secretory pathway. Activated PLCε hydrolyzes PIP_2 into DAG and IP3. DAG activates PKCs. Activated PKCs translocate from the cytosol to the plasma membrane, where they interact with target proteins, resulting in several downstream effects: 1) enhancement of the size of the readily releasable pool of secretory granules, 2) increased Ca^{2+} sensitivity of fusion, and 3) increased Ca^{2+} current at negative potentials.

SUPPORTING MATERIAL

Supporting material can be found online at https://doi.org/10.1016/j.bpj.2024.12.005.

REFERENCES

1. Carmichael, S. W., and H. Winkler. 1985. The adrenal chromaffin cell. *Sci. Am.* 253:40–49.
2. Carbone, E., R. Borges, …, A. Hernández-Cruz. 2019. Chromaffin Cells of the Adrenal Medulla: Physiology, Pharmacology, and Disease. *Compr. Physiol.* 9:1443–1502.
3. Eiden, L. E., and S. Z. Jiang. 2018. What's New in Endocrinology: The Chromaffin Cell. *Front. Endocrinol.* 9:711.
4. Goldstein, D. S. 2010. Adrenal responses to stress. *Cell. Mol. Neurobiol.* 30:1433–1440.
5. Wolf, K., G. Zarkua, …, C. Smith. 2016. Spatial and activity-dependent catecholamine release in rat adrenal medulla under native neuronal stimulation. *Phys. Rep.* 4:e12898.
6. De Robertis, E., and A. V. Ferreira. 1957. Submicroscopic changes of the nerve endings in the adrenal medulla after stimulation of the splanchnic nerve. *J. Biophys. Biochem. Cytol.* 3:611–614.
7. Feldberg, W., B. Minz, and H. Tsudzimura. 1934. The mechanism of the nervous discharge of adrenaline. *J. Physiol.* 81:286–304.
8. Stroth, N., B. A. Kuri, …, L. E. Eiden. 2013. PACAP controls adrenomedullary catecholamine secretion and expression of catecholamine biosynthetic enzymes at high splanchnic nerve firing rates characteristic of stress transduction in male mice. *Endocrinology.* 154:330–339.
9. Smith, C. B., and L. E. Eiden. 2012. Is PACAP the major neurotransmitter for stress transduction at the adrenomedullary synapse? *J. Mol. Neurosci.* 48:403–412.
10. Guerineau, N. C. 2020. Cholinergic and peptidergic neurotransmission in the adrenal medulla: A dynamic control of stimulus-secretion coupling. *IUBMB Life.* 72:553–567.
11. Miyata, A., A. Arimura, …, D. H. Coy. 1989. Isolation of a novel 38 residue-hypothalamic polypeptide which stimulates adenylate cyclase in pituitary cells. *Biochem. Biophys. Res. Commun.* 164:567–574.
12. Chen, X., N. A. Bell, …, A. Anantharam. 2024. A PACAP-activated network for secretion requires coordination of Ca(2+) influx and Ca(2+) mobilization. *Mol. Biol. Cell.* 35:mbcE24020083.
13. Riser, M., and S. D. Norrholm. 2022. Pituitary Adenylate Cyclase Activating Peptide and Post-traumatic Stress Disorder: From Bench to Bedside. *Front. Psychiatr.* 13:861606.
14. Ressler, K. J., K. B. Mercer, …, V. May. 2011. Post-traumatic stress disorder is associated with PACAP and the PAC1 receptor. *Nature.* 470:492–497.
15. Hamelink, C., O. Tjurmina, …, L. E. Eiden. 2002. Pituitary adenylate cyclase-activating polypeptide is a sympathoadrenal neurotransmitter involved in catecholamine regulation and glucohomeostasis. *Proc. Natl. Acad. Sci. USA.* 99:461–466.
16. Taupenot, L., M. Mahata, …, D. T. O'Connor. 1999. Time-dependent effects of the neuropeptide PACAP on catecholamine secretion : stimulation and desensitization. *Hypertension.* 34:1152–1162.
17. Morales, A., R. Mohan, …, A. Anantharam. 2023. PACAP and acetylcholine cause distinct Ca2+ signals and secretory responses in chromaffin cells. *J. Gen. Physiol.* 155:e202213180.
18. Eiden, L. E., A. C. Emery, …, C. B. Smith. 2018. PACAP signaling in stress: insights from the chromaffin cell. *Pflügers Archiv.* 470:79–88.
19. Chen, X., B. L. Coffman, …, A. Anantharam. 2023. Phospholipase C-epsilon defines a PACAP-stimulated pathway for secretion in the chromaffin cell. *J. Neuroendocrinol.* 35:e13255.
20. Smrcka, A. V., J. H. Brown, and G. G. Holz. 2012. Role of phospholipase Cε in physiological phosphoinositide signaling networks. *Cell. Signal.* 24:1333–1343.
21. Wang, H., E. A. Oestreich, …, A. V. Smrcka. 2005. Phospholipase C epsilon modulates beta-adrenergic receptor-dependent cardiac contraction and inhibits cardiac hypertrophy. *Circ Res.* 97:1305–1313.
22. Smith, C., T. Moser, …, E. Neher. 1998. Cytosolic Ca2+ acts by two separate pathways to modulate the supply of release-competent vesicles in chromaffin cells. *Neuron.* 20:1243–1253.
23. Dries, D. R., L. L. Gallegos, and A. C. Newton. 2007. A single residue in the C1 domain sensitizes novel protein kinase C isoforms to cellular diacylglycerol production. *J. Biol. Chem.* 282:826–830.
24. Giovannucci, D. R., M. D. Hlubek, and E. L. Stuenkel. 1999. Mitochondria regulate the Ca(2+)-exocytosis relationship of bovine adrenal chromaffin cells. *J. Neurosci.* 19:9261–9270.
25. Giovannucci, D. R., and E. L. Stuenkel. 1997. Regulation of secretory granule recruitment and exocytosis at rat neurohypophysial nerve endings. *J. Physiol.* 498:735–751.
26. Yang, Y., S. Udayasankar, …, K. D. Gillis. 2002. A highly Ca2+-sensitive pool of vesicles is regulated by protein kinase C in adrenal chromaffin cells. *Proc. Natl. Acad. Sci. USA.* 99:17060–17065.
27. Brose, N., A. Betz, and H. Wegmeyer. 2004. Divergent and convergent signaling by the diacylglycerol second messenger pathway in mammals. *Curr. Opin. Neurobiol.* 14:328–340.
28. Takai, Y., K. Kaibuchi, …, M. Hoshijima. 1985. Role of protein kinase C in transmembrane signaling. *J. Cell. Biochem.* 29:143–155.
29. Kishimoto, A., Y. Takai, …, Y. Nishizuka. 1980. Activation of calcium and phospholipid-dependent protein kinase by diacylglycerol, its possible relation to phosphatidylinositol turnover. *J. Biol. Chem.* 255:2273–2276.
30. de Jong, A. P. H., M. Meijer, …, M. Verhage. 2016. Phosphorylation of synaptotagmin-1 controls a post-priming step in PKC-dependent presynaptic plasticity. *Proc. Natl. Acad. Sci. USA.* 113:5095–5100.
31. Nagy, G., U. Matti, …, J. B. Sørensen. 2002. Protein kinase C-dependent phosphorylation of synaptosome-associated protein of 25 kDa at Ser187 potentiates vesicle recruitment. *J. Neurosci.* 22:9278–9286.
32. Gillis, K. D., R. Mossner, and E. Neher. 1996. Protein kinase C enhances exocytosis from chromaffin cells by increasing the size of the readily releasable pool of secretory granules. *Neuron.* 16:1209–1220.
33. Tewson, P. H., S. Martinka, …, A. M. Quinn. 2016. New DAG and cAMP Sensors Optimized for Live-Cell Assays in Automated Laboratories. *J. Biomol. Screen.* 21:298–305.
34. Lingle, C. J., P. L. Martinez-Espinosa, …, E. Carbone. 2018. Roles of Na(+), Ca(2+), and K(+) channels in the generation of repetitive firing and rhythmic bursting in adrenal chromaffin cells. *Pflügers Archiv.* 470:39–52.
35. Miesenbock, G., D. A. De Angelis, and J. E. Rothman. 1998. Visualizing secretion and synaptic transmission with pH-sensitive green fluorescent proteins. *Nature.* 394:192–195.
36. Rao, T. C., D. R. Passmore, …, A. Anantharam. 2014. Distinct fusion properties of synaptotagmin-1 and synaptotagmin-7 bearing dense core granules. *Mol. Biol. Cell.* 25:2416–2427.
37. Marcantoni, A., D. H. F. Vandael, …, E. Carbone. 2010. Loss of Cav1.3 channels reveals the critical role of L-type and BK channel coupling in pacemaking mouse adrenal chromaffin cells. *J. Neurosci.* 30:491–504.
38. Steinberg, S. F. 2008. Structural basis of protein kinase C isoform function. *Physiol. Rev.* 88:1341–1378.
39. Johannes, F. J., J. Prestle, …, K. Pfizenmaier. 1994. PKCu is a novel, atypical member of the protein kinase C family. *J. Biol. Chem.* 269:6140–6148.
40. Gubernator, N. G., H. Zhang, …, D. Sames. 2009. Fluorescent false neurotransmitters visualize dopamine release from individual presynaptic terminals. *Science.* 324:1441–1444.
41. Kuri, B. A., S. A. Chan, and C. B. Smith. 2009. PACAP regulates immediate catecholamine release from adrenal chromaffin cells in an activity-dependent manner through a protein kinase C-dependent pathway. *J. Neurochem.* 110:1214–1225.

42. Przywara, D. A., X. Guo, …, A. R. Wakade. 1996. A non-cholinergic transmitter, pituitary adenylate cyclase-activating polypeptide, utilizes a novel mechanism to evoke catecholamine secretion in rat adrenal chromaffin cells. *J. Biol. Chem.* 271:10545–10550.

43. Isobe, K., T. Nakai, and Y. Takuwa. 1993. Ca(2+)-dependent stimulatory effect of pituitary adenylate cyclase-activating polypeptide on catecholamine secretion from cultured porcine adrenal medullary chromaffin cells. *Endocrinology.* 132:1757–1765.

44. Newton, A. C. 2001. Protein kinase C: structural and spatial regulation by phosphorylation, cofactors, and macromolecular interactions. *Chem. Rev.* 101:2353–2364.

45. TerBush, D. R., and R. W. Holz. 1990. Activation of protein kinase C is not required for exocytosis from bovine adrenal chromaffin cells: the effects of protein kinase C(19-31), Ca/CaM kinase II(291-317), and staurosporin. *J. Biol. Chem.* 265:21179–21184.

46. Pocotte, S. L., R. A. Frye, …, R. W. Holz. 1985. Effects of phorbol ester on catecholamine secretion and protein phosphorylation in adrenal medullary cell cultures. *Proc. Natl. Acad. Sci. USA.* 82:930–934.

47. Voets, T., E. Neher, and T. Moser. 1999. Mechanisms underlying phasic and sustained secretion in chromaffin cells from mouse adrenal slices. *Neuron.* 23:607–615.

48. Gada, K. D., and D. E. Logothetis. 2022. PKC regulation of ion channels: The involvement of PIP(2). *J. Biol. Chem.* 298:102035.

49. Pinheiro, P. S., S. Houy, and J. B. Sørensen. 2016. C2-domain containing calcium sensors in neuroendocrine secretion. *J. Neurochem.* 139:943–958.

50. Nagy, G., K. Reim, …, J. B. Sørensen. 2004. Regulation of releasable vesicle pool sizes by protein kinase A-dependent phosphorylation of SNAP-25. *Neuron.* 41:417–429.

51. Shimazaki, Y., T. Nishiki, …, M. Takahashi. 1996. Phosphorylation of 25-kDa synaptosome-associated protein. Possible involvement in protein kinase C-mediated regulation of neurotransmitter release. *J. Biol. Chem.* 271:14548–14553.

52. Yang, Y., T. J. Craig, …, K. D. Gillis. 2007. Phosphomimetic Mutation of Ser-187 of SNAP-25 Increases both Syntaxin Binding and Highly Ca2+-sensitive Exocytosis. *J. Gen. Physiol.* 129:233–244.

53. Nili, U., H. de Wit, …, U. Ashery. 2006. Munc18-1 phosphorylation by protein kinase C potentiates vesicle pool replenishment in bovine chromaffin cells. *Neuroscience.* 143:487–500.

54. Montiel, C., I. Mendoza, …, A. M. Cárdenas. 2003. Distinct protein kinases regulate SNAP-25 expression in chromaffin cells. *J. Neurosci. Res.* 71:353–364.

55. Staal, R. G. W., A. Hananiya, and D. Sulzer. 2008. PKC theta activity maintains normal quantal size in chromaffin cells. *J. Neurochem.* 105:1635–1641.

56. Park, Y. S., E. M. Hur, …, K. T. Kim. 2006. Involvement of protein kinase C-epsilon in activity-dependent potentiation of large dense-core vesicle exocytosis in chromaffin cells. *J. Neurosci.* 26:8999–9005.

57. Sena, C. M., L. M. Rosário, …, M. R. Boarder. 1996. Differential regulation of histamine- and bradykinin-stimulated phospholipase C in adrenal chromaffin cells: evidence for involvement of different protein kinase C isoforms. *J. Neurochem.* 66:1086–1094.

58. Starr, E. R., and J. F. Margiotta. 2017. Pituitary adenylate cyclase activating polypeptide induces long-term, transcription-dependent plasticity and remodeling at autonomic synapses. *Mol. Cell. Neurosci.* 85:170–182.

Biophysical Journal, Volume *124*

Supplemental information

Roles for PKC signaling in chromaffin cell exocytosis

Xiaohuan Chen, Nicole A. Bell, Breanna L. Coffman, David R. Giovannucci, and Arun Anantharam

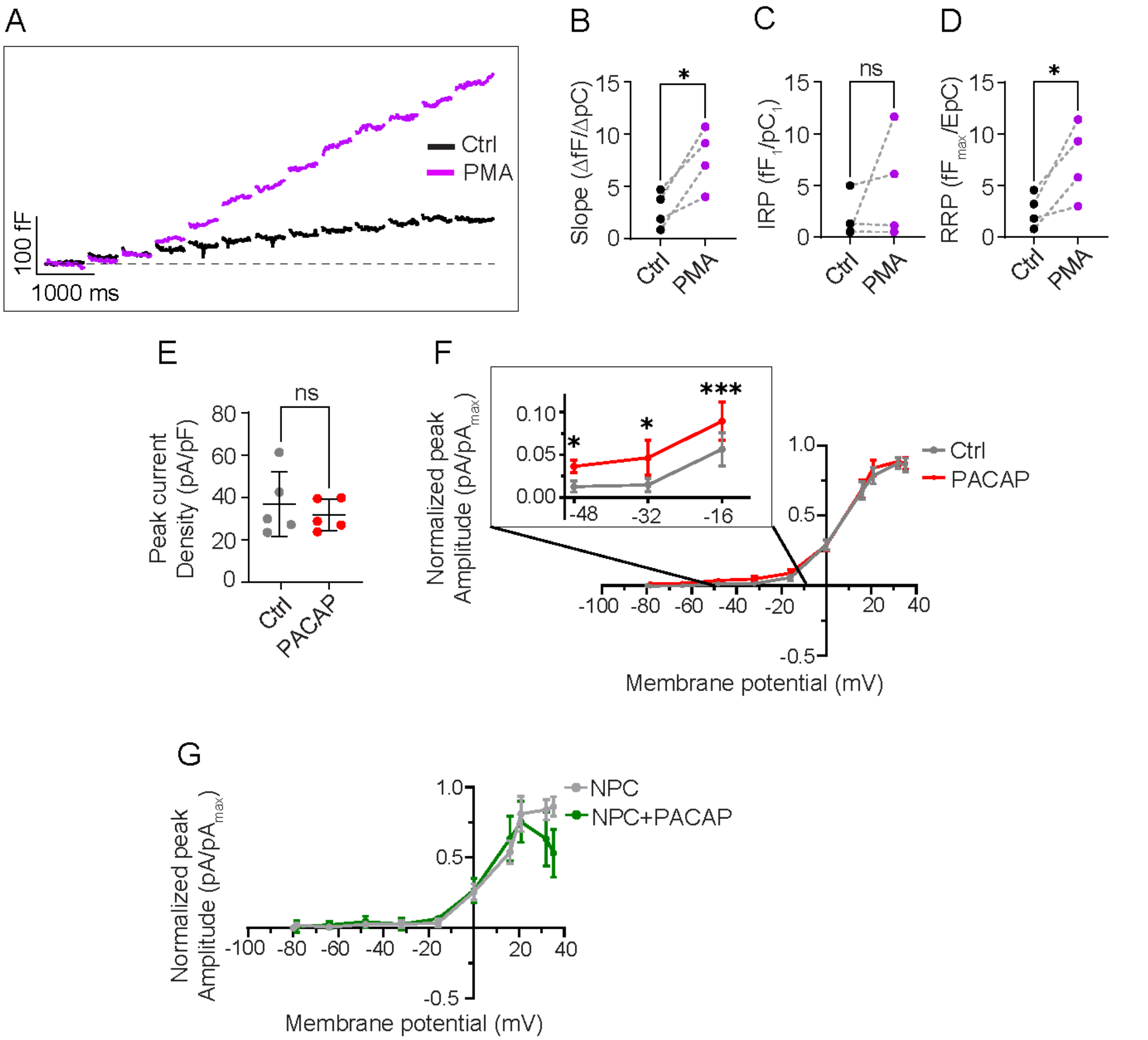

Figure S1: Electrophysiological studies on the effects of PMA and PACAP on chromaffin cell excitability and secretion.

PKC effects on activity-dependent exocytosis were measured using the previously described capacitance paradigm to estimate Ca^{2+} sensitivity, IRP and RRP. PMA was bath applied for 1 minute following a control train and maintained during the second pulse train (A) An example trace of measured capacitance changes (ΔC_m) between a control (black trace) and the PKC

agonist phorbol 12-myristate 13-acetate (PMA) (0.1 μM) (purple trace) treated cell. (B) A comparison of the $pC/\Delta C_m$ relationship between cells pretreated with or without PMA (0.1 μM). Ca^{2+} sensitivity of exocytosis appeared enhanced between the control and PMA (0.1 μM) treated cells ($p = 0.0192$). (C) There was no change in IRP size of PMA treated cells ($p= 0.6$). (D) There was a significant increase in the RRP measured for PMA-treated cells ($p = 0.04$) (n=4). (E) To determine whether PACAP caused an enhancement in Ca^{2+} entry, the peak current density (pA/pF) of Ca^{2+} currents was measured in response to a 50 ms step-depolarization from -80mV to +20mV prior to and following PACAP treatment. Incubation of PACAP (0.5μM) for 1 min did not significantly affect the Ca^{2+} entry ($p = 0.3$) (n=5). (F) To assess PACAP effects on Ca^{2+}channel activation, i-v relationships were measured using a voltage ramp protocol prior to and following PACAP treatment. Following transformation of ramp duration to changes in membrane voltage, the average Ca^{2+} current response to voltage ramp-depolarization from -80mV to +80mV for 100 ms intervals in chromaffin cells were normalized to respective peak amplitude (pA/pA_{max}) and compared. The averaged data showed a modest PACAP-mediated enhancement Ca^{2+} current at membrane potentials of -48 mV ($p = 0.03$), -32 mV ($p = 0.02$), and -16 mV ($p = 0.0005$). Cells were incubated in PACAP (0.5μM) for 1 min prior to test ramp application (n=12). (G) To assess if PACAP-mediated effects on Ca^{2+} channels was dependent on PKC activity, NPC (10μM) was applied for 1 min prior to the first voltage ramp depolarization, followed by a 1 min application of both PACAP (0.5μM) and NPC (10μM) applied together for the second voltage ramp depolarization in the same manner as previously described. Current responses were normalized to peak amplitude (pA/pA_{max}) and demonstrated that PACAP-mediated enhancements at -48 mV ($p = 0.2$), -32 mV ($p = 0.2$), and -16 mV ($p = 0.5$) were blocked by NPC (n=4).

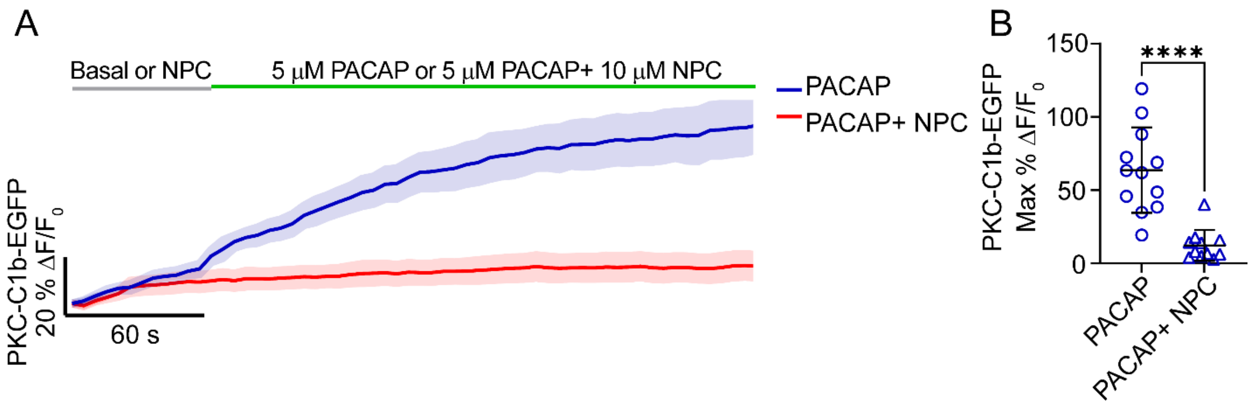

Figure S2. NPC 15437 inhibited PACAP-stimulated translocation of PKCβ C1b-EGFP to the plasma membrane. (A) Time course of PKCβ C1b-EGFP fluorescence changes (%$\Delta F/F_0$) in chromaffin cells stimulated with PACAP (5 µM) alone (blue) or PACAP + NPC 15437 (NPC, 10 µM; red). Cells were pre-incubated in either a basal solution (physiological saline) or with 10 µM NPC for 1 minute, followed by stimulation with PACAP or PACAP + NPC for 4 minutes. Bold lines represent mean fluorescence changes, and shaded areas indicate SEM. (B) Scatter plot showing the maximum %$\Delta F/F_0$ for individual cells under each condition. Data are derived from two independent experiments (PACAP: n = 12 cells; PACAP + NPC: n = 13 cells). Statistical analysis was performed using a two-tailed unpaired t-test, with statistical significance denoted as ****$p < 0.0001$.

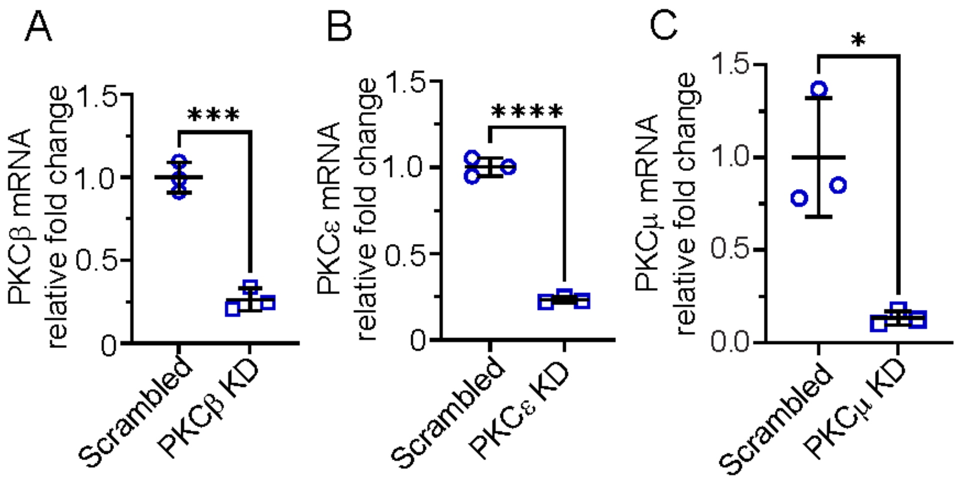

Figure S3. qPCR analysis shRNA knockdown efficiency. qPCR results confirm that shRNAs targeting PKCβ, PKCε, and PKCμ effectively reduce mRNA expression levels by at least 70% for each isoform. Specifically, (A) PKCβ knockdown reduces mRNA by 73%, (B) PKCε by 77%, and (C) PKCμ by 87%. Data are presented as relative fold change compared to scrambled shRNA controls.
